# Expression of THOP1 and Its Relationship to Prognosis in Non-Small Cell Lung Cancer

**DOI:** 10.1371/journal.pone.0106665

**Published:** 2014-09-02

**Authors:** Lei Qi, Shu-hai Li, Li-bo Si, Ming Lu, Hui Tian

**Affiliations:** Department of Thoracic Surgery, Qi Lu Hospital, Shandong University, Jinan, Shandong Province, China; H. Lee Moffitt Cancer Center & Research Institute, United States of America

## Abstract

**Background:**

The study was designed to detect the expression level of thimet oligopeptidase (THOP1) protein in non-small cell lung cancer (NSCLC) and investigate its correlation with clinicopathologic features and prognosis.

**Methods:**

Immunohistochemical staining was used to determine the expression of THOP1 protein in 120 NSCLC specimens and 53 distant normal lung tissues. Quantitative real-time PCR and western blotting were employed to measure the expression of THOP1 in 16 pairs of primary NSCLC and corresponding normal tissues.

**Results:**

Analysis of immunohistochemical staining suggested low THOP1 expression was found in 71 (59.2%) of the 120 NSCLC specimens and significantly correlated with positive lymph node metastasis (*P* = 0.048). However, low THOP1 expression was found in 22 (41.5%) of the 53 normal lung tissues. Chi-square test suggested that the expression of THOP1 was significantly higher in the normal lung tissues than that in the NSCLC specimens (*P* = 0.032). Real-Time PCR and western blotting showed that NSCLC specimens had decreased THOP1 mRNA and protein expression compared to corresponding normal tissues. Univariate analysis demonstrated that low THOP1 expression significantly predicted decreased 5-year disease-free survival (*P* = 0.038) and overall survival (*P* = 0.017). In addition, positive lymph node metastasis (*P* = 0.025) and advanced TNM stage (*P* = 0.009) significantly predicted decreased 5-year overall survival. However, multivariate Cox regression analysis showed that only low THOP1 expression retained its significance as an independent prognostic factor for unfavorable 5-year disease-free survival (*P* = 0.046) and overall survival (*P* = 0.021).

**Conclusions:**

THOP1 may have clinical potentials to be employed as a promising biomarker to identify individuals with better prognosis and a novel antitumor agent for therapy of patients with NSCLC.

## Introduction

Non-small cell lung cancer (NSCLC) is one of the most common intrathoracic malignancies throughout the world due to its late clinical presentation and rapid progression [1], [2]. Despite some advances in early detection and radical surgical resection combined with adjuvant therapy over the recent decade, the prognosis of NSCLC patients remain relatively poor [3]. It is generally acknowledged that genetic heterogeneity and environmental factors lead to tumorigenesis of lung cancer simultaneously. Therefore, identification of novel prognostic biomarkers with possible therapeutic and prognostic relevance is important to optimize the treatment of NSCLC.

Thimet oligopeptidase (THOP1; EC3.4.24.15 or EP 24.15) is a characteristic metallopeptidases with a HEXXH zinc binding motif and associated with metabolism of several neuropeptides containing from 5 to 17 amino acids, such as bradykinin, gonadotropin releasing hormone, opioids and neurotensin [4], [5], [6]. Accumulated evidence suggested that THOP1 was predominately expressed in the cytosol, endoplasmic reticulum, mitochondria and nucleus of different normal human tissues and tumor cells [7], [8], [9]. Moreover, immunohistochemical analysis indicated that THOP1 was present in 90% of NSCLC cell line H1299 and secreted by the cells to cleave and activate DTS-201 [10]. Recent study demonstrated that high THOP1 mRNA expression level in the normal background liver of hepatocellular carcinoma (HCC) was significantly correlated with better survival [11]. Furthermore, THOP1 could inhibit tumor angiogenesis and tumor proliferation promoted by BK in melanoma cells [12]. These findings indicate THOP1 may be a potential anti-tumor agent for tumor therapy.

Expression of THOP1 and its role in prognosis of NSCLC has not been investigated so far. Therefore, we detected expression levels of THOP1 protein in patients with NSCLC by employing immunohistochemical method to investigate its correlation with clinicopathologic features, postoperative tumor relapse and prognosis.

## Materials and Methods

### Ethics Statement

This study was approved by the Ethics Committee of Qilu Hospital. Written informed consent was obtained from each patient to publish the case details, and acquisition of tissue specimens was carried out as prescribed by the institutional guidelines.

### Patients

Tumor samples (n = 120, mean age 61±4.35 years) and distant normal lung tissues (n = 53, 5 cm from the margin of the tumor) were collected from patients with NSCLC who underwent complete tumor resection (lobectomy or pneumonectomy) with regional lymph node dissection between January 2006 and December 2007 at the department of Thoracic Surgery, Qilu Hospital. Histologic examination and grade of cancer cell differentiation were based on the classification system of World Health Organization revised in 2004 and TNM staging system of UICC 2009. No patients had undergone incomplete resection and received chemotherapy or radiation therapy before surgery. The clinical characteristics of these 120 patients are presented in Table 1.

**Table 1:** Correlation of clinicopathologic variables with THOP1 protein in NSCLC.

Category	No. of patients	THOP1		
		high	low	*P* a
Age				0.539
<60 years	67	29	38	
≥60 years	53	20	33	
Sex				0.611
Male	67	26	41	
Female	53	23	30	
Smoking history				0.300
Yes	52	24	28	
No	68	25	43	
Histology				0.826
SCC	70	28	42	
Adeno	50	21	29	
Differentiation				0.247
Well	31	8	23	
Moderate	58	24	34	
Poor	31	17	14	
Tumor size				0.117
≤3 cm	51	25	26	
>3 cm	69	24	45	
Lymph node metastasis				0.048
N0	60	29	29	
N1, N2	73	20	42	
TNM stage				0.491
Stage I	47	21	26	
Stage II and III	73	28	45	

a Chi-square test.

SCC squamous cell cancer.

Adeno adenocarcinoma.

### Immunohistochemistry

Immunohistochemical staining for THOP1 was carried out using the streptavidin-peroxidase method. In brief, 4-µm-thick sections were cut from paraffin-embedded blocks, deparaffinized in xylene and dehydrated through a series of graded alcohol. The sections were incubated in citrate buffer (pH 6.0) with a microwave oven. After be cooled to room temperature, the slides were incubated in 3% hydrogen peroxide for 15 min to block endogenous peroxidase activity and incubated with a mouse monoclonal antibody against THOP1 (Abcam, Cambridge, MA, USA, dilution 1∶50) overnight at 4°C. Subsequently, the biotinylated secondary antibody and peroxidase-conjugated streptavidin complex reagent were applied, followed by counterstaining with Mayer’s haematoxylin. Positive and negative controls were included in each step.

### Evaluation of THOP1 Protein Expression

The immunostained slides were evaluated by two independent investigators in a blinded fashion and reevaluated by these investigators under a multihead microscope in discordant cases to reach a consensus. Expression of the THOP1 protein were evaluated by calculating a total immunostaining score as the product of both the intensity score (0, negative staining; 1, weak staining; 2, moderate staining; 3, strong staining) and proportion score (0, 0–5%; 1, 6–25%; 2, 26–50%; 3, 51–75%; 4, ≥76%), respectively. Thus, the total score ranged from 0 to 7. The cutoff value for high and low THOP1 expression was determined based on a heterogeneity value measured through a log-rank statistical analysis with respect to overall survival. The percentile that yielded the minimum *P* value was chosen as cutoff point [13]. In brief, the 120 patients were arranged in order of the total staining score detected by immunohistochemical analysis from big to small. At each percentile from 10 to 90, the data were divided into two groups and the differences in overall survival were analyzed using the log-rank test. The 41st percentile corresponding to the total staining score of 4 yielded the minimum *P* value (*P* = 0.017) and was chosen as the cutoff point. Therefore, the total staining score of 4 was chosen as the cutoff point for the discrimination between high and low THOP1 expression. Tumors with a total staining score ≥4 were all defined as high THOP1 expression, and tumors with a total staining score <4 were all defined as low THOP1 expression.

### RNA Extraction, Reverse-Transcription Polymerase Chain Reaction and Quantitative Real-Time PCR

Total RNAs were extracted from tissues by using the Trizol (Invitrogen, Carlsbad, CA, USA). Complementary DNA (cDNA) was synthesized by SuperScript III First-Strand Synthesis System (Invitrogen, Carlsbad, CA, USA). Quantitative real-time PCR was carried out by using SYBR Green Supermix (Bio-Rad, CA, USA) for THOP1 according to the manufacturer’s instructions. The levels of THOP1 messenger RNA (mRNA) were normalized to the human β-actin expression level and calculated using the 2^(−△△CT)^ method. Primers were used as the following: sense: The primers for β-actin were: 5′-GCATCCACGAAACTACCT-3′ (forward) and 5′-GAAAGGGTGTAACGCAAC-3′ (reverse). THOP1 primers were: 5′-CAT GGC CAA GAC CAG CCA GA-3′ (forward) and 5′-CGC ACG CTT CAG CTC CAG AA-3′ (reverse).

### SDS-PAGE and Western Blot

Proteins were extracted using RIPA lysis buffer. Equal amounts (20 µg) of protein were subjected to SDS-PAGE analysis, transferred onto nitrocellulose membranes and probed with primary antibodies. Antibody against THOP1 was purchased from Abcam (Abcam, Cambridge, MA, USA, dilution 1∶1000). Anti-β-actin antibody was purchased from Sigma (Sigma, St. Louis, USA). Protein bands were detected using the enhanced chemiluminescence method (Millipore, Billerica, MA, USA). The optical band density was quantified (Imager of Alpha Corporation, San Leandro).

### Follow up

All patients discharged from hospital were followed-up at the outpatient clinic every 3 to 6 months. Follow-up evaluation of patients consisted of physical examination, blood tests, computed tomography, ultrasound examination, chest X-ray, and fiberoptic bronchoscopy if necessary. The location and time of tumor relapse were recorded. Follow-up was completed in all patients until December 2013, and the median follow-up period was 48 months (range: 15∼96 months).

### Statistical Analysis

Statistical analysis of data was performed by SPSS 17.0 system. Chi-square test was performed to examine the association of THOP1 and various clinicopathologic factors. Follow-up time was censored if the patient was lost to follow-up. Survival curves were drawn using the Kaplan–Meier method and compared by the log-rank test. Multivariate Cox regression analysis was used to identify significant independent prognostic factors. All data are mean ± standard deviation (SD) from independent assays. Other statistical analyses were performed using a Student’s t-test. *P*<0.05 was considered to be statistically significant.

## Results

### 1. Expression of THOP1 in NSCLC and its correlation with clinicopathologic factors

**Figure 1:**
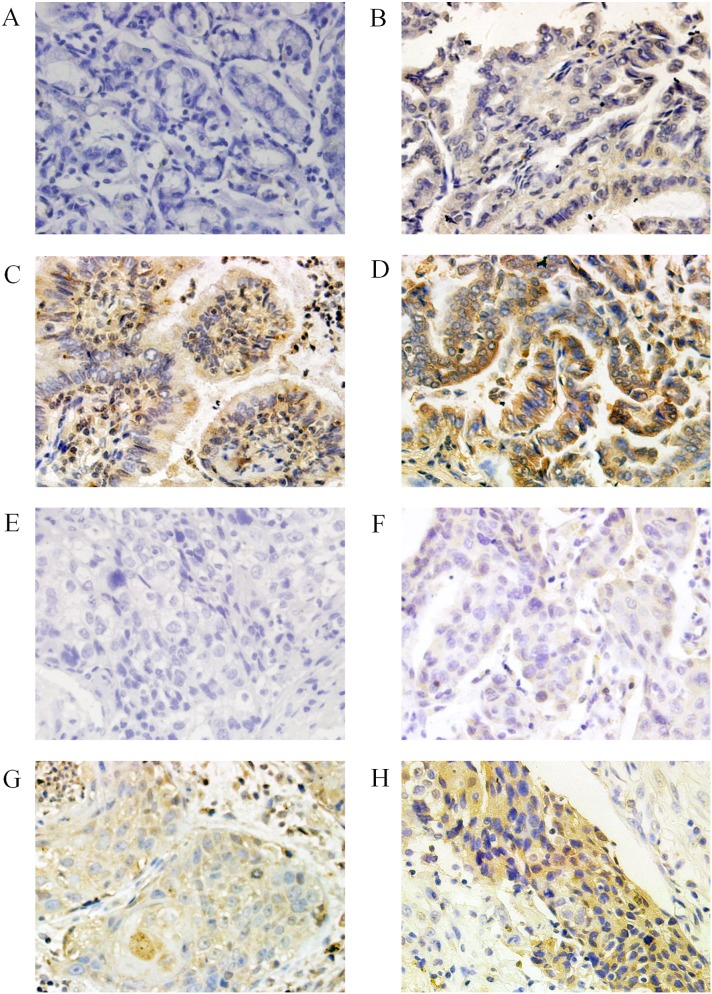
Immunohistochemical staining for THOP1 in NSCLC specimens. Various intensities of THOP1 protein expression in adenocarcinoma, negative expression (A), with weak (B), moderate (C), and strong (D) expression. Various intensities of THOP1 protein expression in squamous cell cancer, negative expression (E), with weak (F), moderate (G), and strong (H) expression.

**Figure 2:**
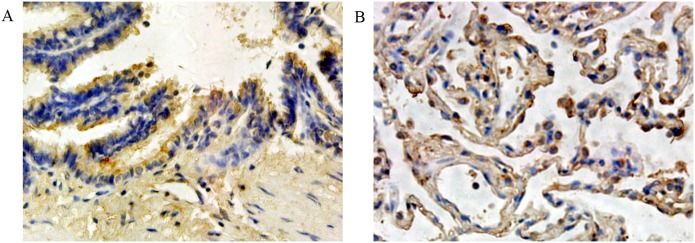
THOP1 was highly expressed in the cytoplasm of normal bronchial epithelium cells (A) and pneumocytes (B).

We detected the expression of THOP1 protein in 120 NSCLC specimens and 53 normal lung tissues using immunohistochemistry. As shown in Fig. 1, immunohistochemistry with THOP1 antibodies showed a positive reaction in the cytoplasm of NSCLC cells. According to our earlier defined criteria, high THOP1 expression was found in 49 (40.8%) of the 120 tumor specimens and low THOP1 expression was found in 71 (59.2%) of the 120 tumor specimens. In addition, as negative controls, immunostaining without primary antibodies resulted in no staining. As shown in Fig. 2, THOP1 was highly expressed in the cytoplasm of normal bronchial epithelium cells and pneumocytes. High THOP1 expression was found in 31 (58.5%) of the 53 normal lung tissues and low THOP1 expression was found in 22 (41.5%) of the 53 normal lung tissues. The expression of THOP1 was significantly higher in the normal lung tissues than that in the NSCLC specimens (chi-square test, *P* = 0.032).

Relationships between THOP1 protein expression and clinicopathologic factors were examined by chi-square test, and our data showed that lower THOP1 expression was significantly correlated with positive lymph node metastasis (*P* = 0.048). There was no statistical significance in relationships between THOP1 expression and other clinicopathologic variables (*P*>0.05, Table 1).

**Figure 3:**
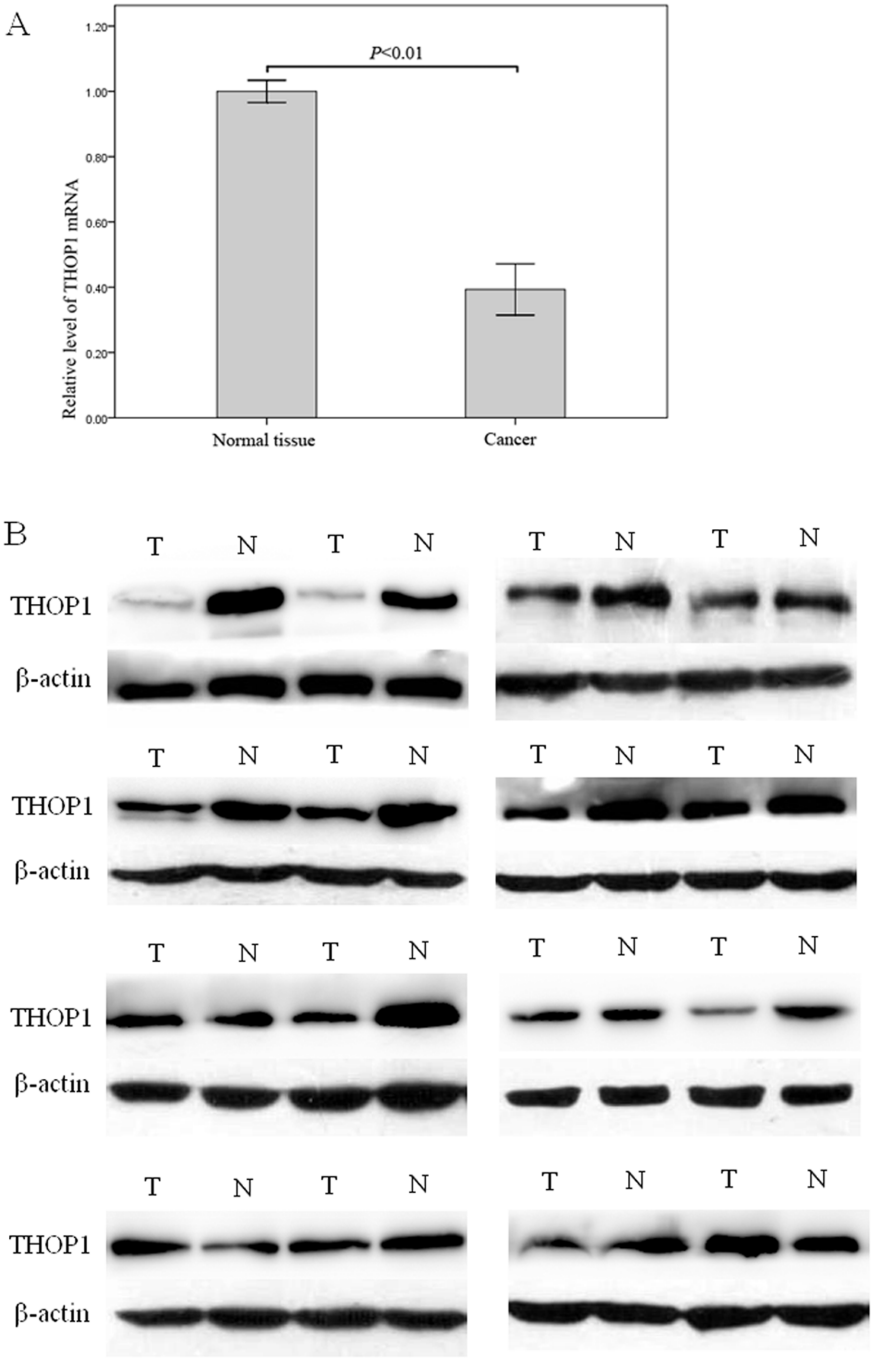
Real-time PCR and Western blot analysis of THOP1 expression in NSCLC. A. Expression of THOP1 mRNA in the NSCLC tissues was lower than that in the normal lung tissues (*P*<0.01). B. Western blot analysis suggested that NSCLC specimens had decreased THOP1 protein expression compared to corresponding normal tissues. N: Normal lung tissue, T: NSCLC specimens.

To investigate the expression of THOP1 gene in NSCLC, we used Quantitative Real-Time PCR to measure the expression of THOP1 mRNA levels in 16 pairs of primary tumors and corresponding normal tissues. Compared to their corresponding normal tissues, NSCLC specimens had decreased THOP1 mRNA expression (Figure 3A). Consistently, western blotting analysis showed that 12 of 16 NSCLC specimens had decreased THOP1 protein expression compared to their corresponding normal tissues (Figure 3B).

### 2. Univariate and multivariate survival analysis for 5-year disease-free survival

**Figure 4:**
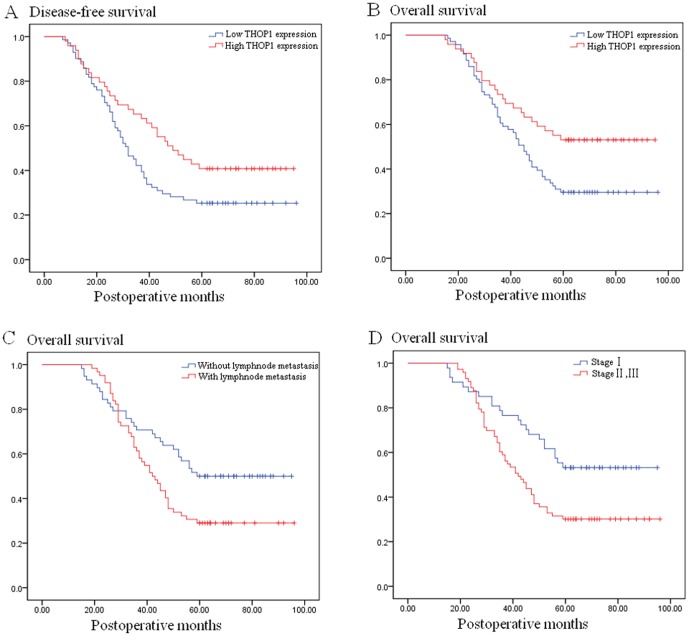
Kaplan–Meier curves of disease-free and overall survival stratified according to THOP1 protein, lymph node metastasis and TNM stage.

Tumor relapse developed within the follow-up period in 82 (68.3%) of the 120 patients: local recurrence in 18 patients, distant metastasis in 52 patients, and both local recurrence and distant metastasis in 12 patients. Univariate analysis (log-rank test) demonstrated that low THOP1 expression (25.4% versus 40.8%, *P* = 0.038, Figure 4A) significantly predicted decreased 5-year disease-free survival. Moreover, the results of multivariate Cox regression analysis showed that low THOP1 expression retained its significance as an independent prognostic factor for unfavorable 5-year disease-free survival (*P* = 0.046, Table 2).

**Table 2:** Univariate and multivariate analysis for disease-free survival.

Variable	Univariate analysis	Multivariate analysis		
	*P*	95% CI	Exp(B)	*P*
Sex (Male vs Female)	0.317	0.790–1.963	1.245	0.346
Age (<60 years vs ≥60 years)	0.725	0.527–1.318	0.833	0.435
Smoking (Yes vs No)	0.791	0.780–2.058	1.267	0.338
Histology (SCC vs Adeno)	0.284	0.789–1.977	1.249	0.343
Differentiation (Poor vs Moderate vs Well)	0.763	0.743–1.411	1.024	0.884
Tumor size (≤3 cm vs >3 cm)	0.061	0.822–2.141	1.327	0.247
Lymph node metastasis (N0 vs N1 and N2)	0.092	0.630–2.694	1.303	0.475
TNM (Stage I vs Stage II and III)	0.109	0.514–2.344	1.097	0.810
THOP1 (High vs Low)	0.038	0.366–0.990	0.602	0.046

CI confidence interval.

SCC squamous cell cancer.

Adeno adenocarcinoma.

### 3. Univariate and multivariate survival analysis for 5-year overall survival

Of the 120 patients, 73 (60.8%) cases died of cancer-related causes within 5 years after operation, the 5-year overall survival was 39.2%. Univariate analysis (log-rank test) demonstrated that low THOP1 expression (29.6% versus 53.1%, *P* = 0.017, Figure 4B), positive lymph node metastasis (29.0% versus 50.0%, *P* = 0.025, Figure 4C) and advanced TNM stage (30.1% versus 53.2%, *P* = 0.009, Figure 4D) significantly predicted poor 5-year overall survival (Table 3). The results of multivariate Cox regression analysis showed that the significant prognostic effect of positive lymph node metastasis (*P* = 0.653, Table 3) and advanced TNM stage (*P* = 0.218, Table 3) observed on univariate analysis disappeared, and only low THOP1 expression retained its significance as an independent prognostic factor for unfavorable overall survival (*P* = 0.021, Table 3).

**Table 3:** Univariate and multivariate analysis for overall survival.

Variable	Univariate analysis	Multivariate analysis		
	*P*	95% CI	Exp(B)	*P*
Sex (Male vs Female)	0.993	0.632–1.633	1.016	0.947
Age (<60 years vs ≥60 years)	0.708	0.604–1.558	0.970	0.900
Smoking (Yes vs No)	0.845	0.881–2.394	1.452	0.144
Histology (SCC vs Adeno)	0.189	0.820–2.136	1.324	0.251
Differentiation (Poor vs Moderate vs Well)	0.866	0.789–1.574	1.114	0.539
Tumor size (≤3 cm vs >3 cm)	0.070	0.768–2.118	1.275	0.348
Lymph node metastasis (N0 vs N1 and N2)	0.025	0.557–2.545	1.191	0.653
TNM (Stage I vs Stage II and III)	0.009	0.735–3.854	1.683	0.218
THOP1 (High vs Low)	0.017	0.312–0.911	0.533	0.021

CI confidence interval.

SCC squamous cell cancer.

Adeno adenocarcinoma.

## Discussion

Although being considered to play a role in the neuroendocrine system [14], THOP1 has become a recent focus in tumor research. Cell fractionation studies have demonstrated that THOP1 is present in the particulate subcellular fractions of the central nervous tissue [15] and secreted from distinctive cell lines, such as the rat glioma C6 [16] and mouse AtT-20 [17]. Moreover, high THOP1 mRNA expression in corresponding normal tissue of hepatocellular carcinoma (HCC) was correlated with a better overall survival [11]. However, few studies have detected the expression of THOP1 protein in tumor tissues and its relationship to tumor prognosis.

In the present study, we investigated the expression of THOP1 in NSCLC and corresponding normal tissues, and then analyzed the relationships between THOP1 expression and other clinicopathologic variables in NSCLC. The findings of our study showed that THOP1 was universally expressed in NSCLC and corresponding normal tissues, and expression of THOP1 in normal tissues of NSCLC was significantly elevated in both mRNA and protein levels. In addition, lower THOP1 expression in NSCLC was significantly correlated with positive lymph node metastasis. Kaplan–Meier analysis in this study showed that low THOP1 expression significantly predicted decreased 5-year disease-free survival and overall survival. In addition, positive lymph node metastasis and advanced TNM stage significantly predicted decreased 5-year overall survival. To eliminate the impact of mixed factors correlated with prognosis on statistical analysis, the Cox regression multivariate analysis was performed to determine independent prognostic factors. Multivariate analysis showed that only THOP1 maintained its prognostic value as independent prognostic factor for poor disease-free and overall survival. Our survival analysis demonstrated that the expression of THOP1 protein could be a useful diagnostic marker for patients with NSCLC. As stated above, our results implied that THOP1 protein may have an antitumor effect in carcinogenesis and tumor progression of NSCLC.

Previous study suggested that THOP1 was able to hydrolyze several kinds of bioactive peptides such as bradykinin (BK) [18]. BK is an important mediator responsible for tumor-associated angiogenesis and tumor growth [19], [20], [21]. In addition, BK was involved in carcinogenesis and cancer progression by increasing α2β1 integrin expression through the BK receptor signal pathway [22]. Moreover, as an antagonist factor to BK, THOP1 could inhibit angiogenesis and tumor growth promoted by BK [12]. Pathological angiogenesis is a relatively early event in the carcinogenesis and increased tumor angiogenesis is correlated with tumor invasion, metastasis and poor prognosis [23], [24]. Therefore, decreased expression of THOP1 in NSCLC may be responsible for cancer development, malignancy and poor survival rate due to its reduction in anti-BK function.

Exciting progress has been made in employing THOP1 for immunotherapy in cancers. It was reported that THOP1 secreted by tumor cells was a very likely candidate for the extracellular activation of CPI-0004Na which was a prodrug of doxorubicin, and the improved therapeutic index of CPI-0004Na compared with free doxorubicin was confirmed in three tumor xenograft models of prostate, breast, and lung cancer [10], [25]. Another study about cytotoxic T lymphocytes (CTLs) suggested THOP1 could strengthen the immune defense against intracellular pathogens and cancer by contributing to the C-terminal trimming of CTL epitopes [26]. Therefore, THOP1 may have clinical potential to be employed as a more useful agent of antitumor therapy for patients with NSCLC.

In conclusion, our retrospective analysis provided evidence that the expression of THOP1 was significantly higher in the normal lung tissues than that in the NSCLC specimens, and low THOP1 expression was significantly correlated with positive lymph node metastasis in NSCLC. Moreover, high THOP1 protein expression exhibited antitumor property and showed positive influence on both disease-free and overall survival. Therefore, THOP1 may have clinical potentials to be employed as a promising biomarker to identify individuals with better prognosis and a novel antitumor agent for therapy of patients with NSCLC.
